# Closed laparoscopic and endoscopic cooperative surgery for early gastric cancer with difficulty in endoscopic submucosal dissection: a report of three cases

**DOI:** 10.1186/s40792-020-01015-4

**Published:** 2020-09-29

**Authors:** Hiroshi Saito, Akihiro Nishimura, Yusuke Sakimura, Hiroki Tawara, Kengo Hayashi, Kaichiro Kato, Toshikatsu Tsuji, Daisuke Yamamoto, Hirotaka Kitamura, Shinichi Kadoya, Hiroyuki Bando

**Affiliations:** Department of Gastroenterological Surgery, Ishikawa Prefectural Central Hospital, Kuratsuki-Higashi 2-1, Kanazawa, Ishikawa Japan

**Keywords:** Endoscopic submucosal dissection, Closed laparoscopic and endoscopic cooperative surgery, Early gastric cancer

## Abstract

**Background:**

Endoscopic submucosal dissection (ESD) is increasingly applied for early gastric cancer. ESD is a less invasive procedure and could be a radical treatment. However, in some cases, ESD cannot be completed owing to patient or technical factors. In such cases, which could have the potential for curative resection with ESD, standard gastrectomy is excessively invasive. Through closed laparoscopic and endoscopic cooperative surgery (LECS), gastric tumor can be precisely resected without exposing tumor cells to the abdominal cavity. Compared with standard gastrectomy, closed LECS is less invasive for the treatment of early gastric cancer.

**Case presentation:**

We performed closed LECS for three cases of early gastric cancer after failed ESD. In all three cases, ESD was interrupted owing to technical and patient factors, including perforation, respiratory failure, and carbon dioxide narcosis. All three cases successfully underwent closed LECS with complete tumor resection and showed an uneventful postoperative course. All three patients remain alive and have experienced no complications or recurrence, with a median follow up of 30 (14–30) months.

**Conclusions:**

Closed LECS is less invasive and useful procedure for the treatment of early gastric cancer, particularly in cases with difficulty in ESD.

## Background

Endoscopic submucosal dissection (ESD) has been accepted as a less invasive procedure for local resection of early gastric cancer [1, 2]. The criteria for ESD in early gastric cancer have been expanded to include larger intramucosal or ulcerated lesions [3]. However, ESD remains complicated and some cases have been accompanied by complications leading to interruption of ESD.

Before the development of ESD, some patients with early gastric cancer underwent local resection of the stomach using a lesion-lifting technique [4–7]; however, there were problems in determining the exact dissection line to maintain safe margins.

Laparoscopic and endoscopic cooperative surgery (LECS) for gastric submucosal tumor (SMT) was first reported by Hiki et al. in 2008 [8]; since then, the use of LECS has rapidly increased. However, the classical LECS procedure involves a risk of gastric contents or tumor cells coming into contact with the abdominal cavity. Therefore, this procedure is considered unsafe for early gastric cancer. To prevent this issue, we developed the closed laparoscopic and endoscopic cooperative surgery (LECS) technique. In closed LECS, tumor cells do not come into contact with the abdominal cavity; thus, this procedure may prevent tumor cell dissemination in the abdominal cavity.

Here, we describe three cases of closed LECS for early gastric cancer in which ESD could not be completed previously.

## Case presentation

### Case 1

A 54-year-old male was referred by a local clinic to the previous hospital for the treatment of early gastric cancer. ESD was attempted, but resection line was fibrotic and rich in blood flow; therefore, it was difficult to detach the tumor region. Perforation during the detachment of the submucosal layer interrupted the ESD procedure (Table 1). He was then referred to our hospital for further surgical treatment.

**Table 1:** Patients characteristics of the three patients

	Case 1	Case 2	Case 3
Age	54	74	58
Sex	Male	Male	Female
BMI (kg/m^2^)	22.2	22.3	22.0
Location of tumor	Upper, anterior	Middle, anterior, greater curvature	Upper, posterior, greater curvature
Tumor size (mm)	14	7	7
Cause of ESD interruption	Perforation	Respiratory failure	Carbon dioxide narcosis

**Fig. 1:**
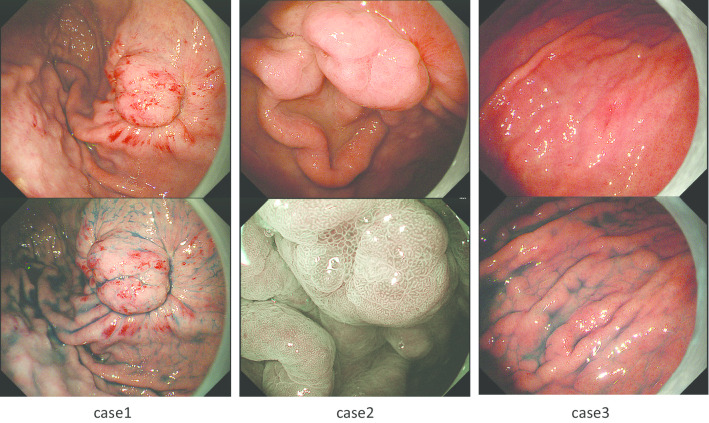
Preoperative endoscopy findings

Endoscopic examination before ESD revealed a class 0-IIc lesion that was 10 mm in diameter and located at the anterior wall of upper gastric body; pathological diagnosis was tubular adenocarcinoma (Fig. 1). Computer tomography revealed no positive lymph nodes or distant metastases.

The operation was performed 53 days after the ESD procedure. Laparoscopy revealed that the lesion was located in the anterior wall of the upper gastric body, and slight adhesion was observed between the perforated site and surrounding tissues. The lesion was easy to detach, and we obtained a clear view for the closed LECS procedure. The operation time was 230 min, and blood loss was during the operation was 10 ml. The postoperative course was uneventful. Meals were started on postoperative day (POD) 3 and he was discharged at 7 PODs (Table 2).

**Table 2:** Surgical outcome

	Case 1	Case 2	Case 3
Operation time (min)	115	125	148
Blood loss (ml)	15	3	15
Intraoperative complication	None	None	None
Postoperative complication	None	None	None
Postoperative hospital stay (days)	7	8	15
Relapse-free survival (months)	30	30	14
Functional complication	None	None	None
Remnant stomach deformity	None	None	None

Pathological examination confirmed the intramucosal location of the tumor, which measured 14 × 5 mm in diameter with no lymphatic or venous invasion. The resected specimen was 47 × 38 mm in diameter with negative lateral and vertical margins. The submucosal layer near the tumor site showed fibrosis, and we observed edematous change and tissue congestion caused by the previous perforation.

The patient visited our outpatient department regularly; however, there were no postoperative functional complications and no findings of remnant stomach deformities on follow-up esophagogastroduodenoscopy (EGD) (Table 2).

### Case 2

A 74-year-old male was referred by a previous hospital to our hospital for the treatment of early gastric cancer. ESD was attempted at the Department of Gastroenterology in our hospital, but the procedure was interrupted owing to respiratory failure (Table 1). He was then referred to our department for surgical treatment.

Endoscopic examination before ESD revealed a 10 mm diameter class 0-IIc lesion that was located at the anterior wall and greater curvature side of the middle gastric body; pathological diagnosis was tubular adenocarcinoma (Fig. 1). No positive lymph nodes or distant metastases were detected on computer tomography.

The operation was performed 35 days after the ESD procedure. The tumor was located in the anterior wall and at the greater curvature of the middle gastric body. No adhesion was seen around the tumor site. The operation time was 125 min, and blood loss during the operation was 3 ml. The postoperative course was uneventful. Meals were started on POD 3 and he was discharged at 8 PODs (Table 2).

Pathological examination confirmed the intramucosal location of the tumor that measured 7 × 5 mm in diameter with no lymphatic or venous invasion. The resected specimen was 47 × 37 mm in diameter with negative lateral and vertical margins.

The patient visited our outpatient department regularly; however, no postoperative functional complications and no findings of remnant stomach deformities were noted on follow-up EGD (Table 2).

### Case 3

A 58-year-old female was referred by a previous clinic to our hospital for the treatment of gastric cancer. ESD was attempted at the Department of Gastroenterology in our hospital, but it could not be completed as the patient experienced carbon dioxide narcosis (Table 1). She was then referred to our department for further surgical treatment.

Endoscopic examination before ESD revealed a 7-mm diameter class 0-IIc lesion located at the posterior wall and greater curvature side of upper gastric body; histological diagnosis was suspected adenocarcinoma (Fig. 1). Computer tomography revealed no lymph nodes or distant metastases.

The operation was performed 60 days after the ESD procedure. The tumor site was located at the posterior wall and greater curvature side of upper gastric body. There was no adhesion around the tumor site. The operation time was 148 min, and blood loss during the procedure was 15 ml. The postoperative course was uneventful, and she was discharged 15 days after surgery (Table 2).

Pathological examination revealed no malignant findings, and the final diagnosis was group 4. The resected specimen was 45 × 34 mm in diameter.

The patient visited our outpatient department regularly; however, there was no postoperative functional complications and no findings of remnant stomach deformities on follow-up EGD (Table 2).

### Procedure of closed LECS (Fig. 2)

After the patients were prepared for laparoscopic surgery, the tumor location was first confirmed using intraluminal endoscopy. The periphery of the tumor was marked to the tumor edge with a margin, and the marked area was resected circumferentially using a needle knife and an IT knife 2 by the endoscopic submucosal dissection technique, and submucosal dissection was performed deeper and wider above the proper muscular layer.

**Fig. 2:**
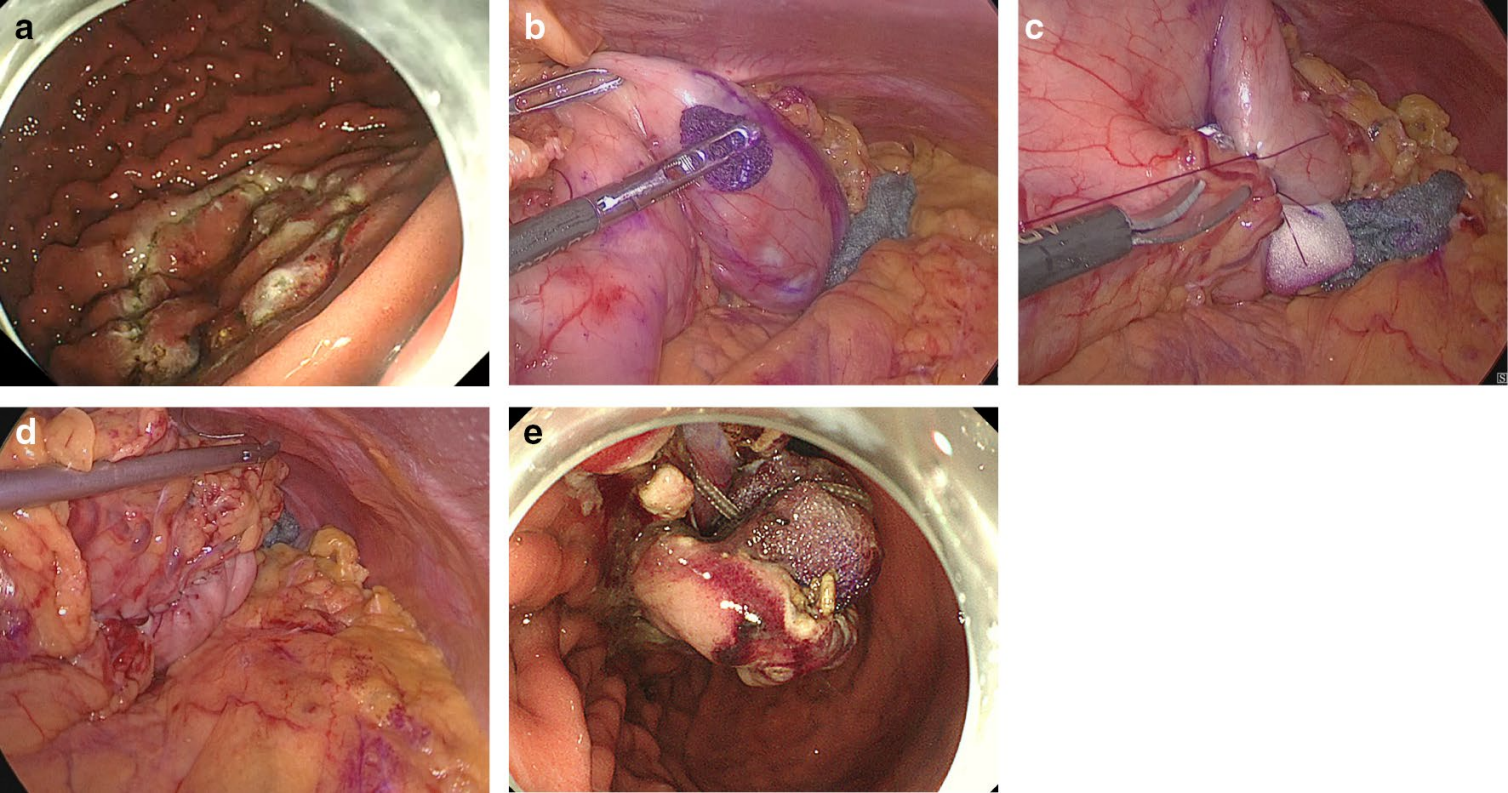
Procedure for closed laparoscopic and endoscopic cooperative surgery. **a** Circumferential endoscopic submucosal resection around the tumor. **b** Laparoscopic serosal marking under guidance by endoscopy. **c**, **d** Seromuscular suture with inversion of the marked lesion into the inside of the stomach in such a way to bury a spongy spacer. **e** Endoscopic seromuscular dissection. The spacer extended the space between the sutured seromuscular plane and the serosal surface of the inverted lesion

The tip of the needle knife remained visible in the laparoscopic image beyond the seromuscular layer. The serosal layer was marked along the dissection line of the mucosal layer. Subsequently, a spongy spacer, Secrea^®^ (Hogy Medical, Tokyo, Japan) was placed on the serosal surface and fixed at the center of the suture line by suturing both sides of the serosa, and seromuscular continuous suturing was performed to bury the spacer using 3–0 absorbable barbed suture. Finally, we performed circumferential seromuscular dissection using endoscopy along the submucosal dissection line. As the full thickness of the wall was cut, the buried Securea spacer was exposed to the luminal side. The specimen and Securea were removed via the oral cavity. Endoscopic insufflation was performed to ensure that there were no air leaks, and the absence of bleeding was confirmed via endoscopy and laparoscopy.

## Discussion

In these three cases, closed LECS resulted in successful resection of early gastric cancer after failed ESD. One essential feature of closed LECS is that tumor cells do not come into contact with or disseminate into the peritoneal cavity. To prevent exposure of tumor cells to the abdominal cavity, the tumor was oriented toward the intra-gastric cavity by seromuscular suture. In our report, one case had a history of perforation during ESD. Ikehara et al. reported [9] that gastric perforation during ESD for gastric cancer does not lead to tumor cell dissemination into the peritoneum even in the long term. Therefore, closed LECS in this case was feasible in terms of oncological viewpoint.

Other gastric wall full-thickness resection techniques such as “CLEAN-NET” or “NEWS” have been developed to prevent the dissemination of tumor cells [10, 11]. CLEAN-NET is a technique for the full-thickness resection of the stomach wall using only laparoscopy; the line of dissection is then confirmed via endoscopy. In contrast, NEWS utilizes endoscopy to assist with a laparoscopic approach. Compared with standard gastrectomy, CLEAN-NET and NEWS are feasible, safe, and less invasive and can be considered as feasible treatment options for patients with early gastric cancer who had difficulties with ESD. However, the mucosal layer considerably shifts from the seromuscular layer during surgery; thus the seromuscular layer may be incorrectly dissected using CLEAN-NET and NEWS [3]. This could lead to selection of an inappropriate resection line for the gastric cancer. Inverted LECS using the crown method is a modified LECS procedure. In this procedure, the tumor is inverted to face the intragastric cavity to prevent contact between the tumor and the visceral tissue. Moreover, the appropriate dissection line was easily identified because of the endoscopic submucosal resection around the tumor and its confirmation both laparoscopically and endoscopically [3].

Considering the indication of closed LECS, there are certain limitations of this procedure. As the resected tumor could not pass through the esophagogastric junction, closed LECS is indicated for tumors ≤ 30 mm in diameter [12]. An inverted LECS procedure may be suitable for the resection of a gastric tumor > 30 mm in size. With regard to tumor location, closed LECS is considered to have no limitations [12]. However, it is believed that lesions that are located very close to the pylorus or cardia are not indicated with closed LECS owing to concerns of postoperative obstruction. Furthermore, Waseda et al. reported [13] that the resection of tumors with lesser curvature may lead to gastric motility disorder. It is associated with the resection of Latarjet’s branch of the vagal nerve and gastric deformity. In our study, there were no postoperative gastric motility disorders in any patient; this could be because postoperative deformities were not highlighted and tumor location was the anterior wall or greater curvature. Closed LECS may not be suitable for tumors with lesser curvature.

When local resection of the stomach for early gastric cancer is performed, it can be difficult to determine a dissection line that provides a safe margin from the tumor using a laparoscopic approach. Laparoscopic local resection of the stomach using the lesion-lifting technique has been performed to treat early gastric cancer [4]; however, there was a relatively high rate of local recurrence after laparoscopic resection of the stomach using the lesion-lifting technique compared with the results of ESD for early gastric cancer [5, 14]. ESD has since become the standard treatment procedure for early gastric cancer. In closed LECS, the resection margins could be determined endoscopically. Moreover, closed LECS combines the ESD technique and laparoscopic gastric wall resection; this prevents excessive resection and deformation of the stomach after surgery [3]. This procedure could be feasible for cases of early gastric cancer that are accompanied by complications causing the interruption of ESD.

## Conclusions

In conclusion, closed LECS could prevent the dissemination of tumor cells within the abdominal cavity and facilitates endoscopic determination of the appropriate resection line. This procedure is a feasible treatment option for early gastric cancers that are difficult to treat with ESD.

## Data Availability

This case report does not have a dataset. All related data are included within the article.

## Abbreviations

ESD: Endoscopic submucosal dissection
LECS: Laparoscopic and endoscopic cooperative surgery
